# Molecular characterization and overexpression of the difenoconazole resistance gene *CYP51* in *Lasiodiplodia theobromae* field isolates

**DOI:** 10.1038/s41598-021-03601-4

**Published:** 2021-12-21

**Authors:** Chenguang Wang, Luxi Xu, Xiaoyu Liang, Jing Wang, Xinwei Xian, Yu Zhang, Ye Yang

**Affiliations:** 1 College of Plant Protection, Hainan University, Haikou, 570228 China; 2 Key Laboratory of Green Prevention and Control of Tropical Plant Diseases and Pests, Ministry of Education, Haikou, China

**Keywords:** Mechanism of action, Plant molecular biology

## Abstract

Stem-end rot (SER) caused by *Lasiodiplodia theobromae* is an important disease of mango in China. Demethylation inhibitor (DMI) fungicides are widely used for disease control in mango orchards. The baseline sensitivity to difenoconazole of 138 *L. theobromae* isolates collected from mango in the field in 2019 was established by the mycelial growth rate method. The cross-resistance to six site-specific fungicides with different modes of action were investigated using 20 isolates randomly selected. The possible mechanism for *L. theobromae* resistance to difenoconazole was preliminarily determined through gene sequence alignment and quantitative real-time PCR analysis. The results showed that the EC_50_ values of 138 *L. theobromae* isolates to difenoconazole ranged from 0.01 to 13.72 µg/mL. The frequency of difenoconazole sensitivity formed a normal distribution curve when the outliers were excluded. Difenoconazole showed positive cross-resistance only with the DMI tebuconazole but not with non-DMI fungicides carbendazim, pyraclostrobin, fludioxonil, bromothalonil, or iprodione. Some multifungicide-resistant isolates of *L. theobromae* were found. Two amino acid substitutions (E209k and G207A) were found in the CYP51 protein, but they were unlikely to be related to the resistance phenotype. There was no alteration in the promoter region of the *CYP51* gene. However, difenoconazole significantly increased the expression of the *CYP51* gene in the resistant isolates compared to the susceptible isolates. These results are vital to develop effective mango disease management strategies to avoid the development of further resistance.

## Introduction

Mango (*Mangifera indica* L.), known as the ‘king of tropical fruits’, is one of the main tropical and subtropical fruits and is widely appreciated for its economic value and high nutritional value^1^. China is the world's second-largest mango grower, being only inferior to India. The main mango-producing area in China is Hainan Province, in which the mango planting area exceeded 56,900 hectares in 2019^2^. The majority of mangoes are intended for fresh-market consumption. Thus, any surface flaws impact fruit sales. In Hainan, *Lasiodiplodia theobromae* is the main pathogen causing stem-end rot (SER) of mango^3^. This fungus may establish itself in the field asymptomatically and stay in a quiescent state. The pathogen develops rapidly after the fruit has been harvested, which causes fruit rot and serious damage to fruit quality during the storage and transportation of mango; this results in huge economic losses^4–6^. The pathogen can also infect various other plants and cause diseases in the field and storage period, including blueberry^7^, coconut^8^, papaya^9^, longan fruit^10^, and so on. Since no cultivars show resistance to *L. theobromae*, SER disease management has depended on chemical control. Fungicide benzimidazole methylcarbamate (MBCs) and sterol 14α-demethylase inhibitors (DMIs) are extensively used to control mango disease ^11,12^.

In Hainan Province, China, many DMI fungicides have been frequently used to control various diseases during mango cultivation placing great pressure on the selection of fungicides for *L. theobromae* and generating the risk of serious resistance of this pathogen to fungicides. To determine the difenoconazole resistance of *L. theobromae* in Hainan and to explore the mechanisms of resistance, the aims of this study were to (I) determine the sensitivity of *L. theobromae* isolates to difenoconazole; (II) identify the patterns of cross-resistance between difenoconazole and other DMIs or fungicides that have different mechanisms of action than difenoconazole; and (III) investigate the molecular mechanisms that may be responsible for difenoconazole resistance.

## Materials and methods

### Isolates and culture conditions

In 2019, 138 single-spore field isolates of *L. theobromae* were obtained from diseased mango fruits in Hainan Province, China, previously identified and preserved in our laboratory. At 28 °C in the dark, the isolates were grown on potato dextrose agar (PDA) medium. All the procedures followed for using the mango fruit comply with relevant institutional, national, and international guidelines and legislation.

### Determination of the baseline sensitivity of field isolates to difenoconazole

A mycelial growth inhibition assay was used to investigate the baseline sensitivity to difenoconazole of 138 *L. theobromae* isolates^13^. To prepare stock solutions, difenoconazole (97.2%; Zhengye Chemical Industrial Co., Hainan, China) was dissolved in 100% acetone to obtain 5 × 10^3^ μg/mL solutions. A mycelial plug (5 mm in diameter) from the edge of the 3-day-old culture of each isolate was inoculated in 90 mm diameter Petri plates containing difenoconazole PDA media. The difenoconazole concentrations were 51.2, 12.8, 3.2, 0.8, and 0.2 μg/mL. The final concentration of acetone solvent in the medium was 0.1%. There was also a control medium with the same amount of acetone but no fungicide. Inoculated plates were cultured in the dark at 28 °C. The diameter of each colony was measured, and the inhibition rate of mycelial development calculated after cultured for 36 h. There were three replicate plates per treatment. The entire experiment was repeated twice independently. The frequency distribution of 138 EC_50_ values of *L. theobromae* was plotted to represent the baseline sensitivity. The baseline sensitivity level of *L. theobromae* was used to develop classification criteria for difenoconazole-sensitive phenotypes^13,14^. The resistance factor (RF) of each isolate to fungicide was computed using the baseline sensitivity: sensitive isolates (S): RF < 5; resistant isolates (R): RF > 5^15^.

### Cross-resistance of difenoconazole with other fungicides

The cross-resistance of difenoconazole with other regularly used fungicides was investigated using 20 isolates. These comprised DMIs fungicides tebuconazole and five fungicides of other action modes, which were carbendazim (benzimidazole), iprodione (dicarboximides), bromothalonil (bromomethyl glutaronitrile), fludioxonil (phenylpyrrole) and pyraclostrobin (strobilurin). As previously stated, EC_50_ values were calculated using a mycelial growth inhibition experiment. The EC_50_ values of the fungicides tested were used to determine cross-resistance correlations. The experiment was conducted three times independently, using three replicate plates for each treatment.

### Cloning and sequencing of *LtCYP51* gene

Mycelia of *L. theobromae* were snap-frozen in liquid nitrogen and processed with tungsten beads in a LUKYM-II Mixer-Mill to extract total genomic DNA from 30 isolates (Guangzhou Luka sequencing instrument Co., LTD, Guangzhou, China). Total genomic DNA was extracted according to the manufacturer's instructions using the E.Z.N.A.^®^ HP Plant DNA Mini kit (Omega Bio-Tek, Norcross, United States). Primer pairs, LtCYP51-F1/LtCYP51-R1 and Per-1F/Per-1F (Table 1), were designed to amplify the *LtCYP51* coding sequence and *LtCYP51* promoter of resistant and sensitive isolates. Amplications were performed in a My Cycler thermal cycler (Bio-Rad Laboratories, Hercules, CA, USA). A 40 µL reaction volume was used for PCR amplification, with 20 µL of 2 × Phanta Max Master Mix, 0.8 µL of template DNA, 1.6 µL of (10 mM) each primer, and 16 µL of ddH2O. The PCR settings for the coding sequence were 95 °C for 3 min, followed by 35 cycles of 95 °C for 30 s, 58 °C for 50 s, and 72 °C for 90 s, followed by a final 5-min extension at 72 °C.

**Table 1:** Primers utilized in this study.

Primers	Sequence (5′ → 3′)	Description
Per-1F	GCCAACAGCCACGGATGAT	Amplification of the promoter region of the Lt CYP51 gene
Per-1R	GCCATAGGTGACGGTGCTG	
Lt-CYP51F	CCCTCCGTCTCCCTACACCT	Amplification of the coding region of the Lt CYP51 gene
Lt-CYP51R	TTCTCCCTCCTCTCCCAAA	
RT-LtF	GGATTGTGCTTCGTCTCGC	Quantitative RT-PCR primers for analysis of Lt CYP51 expression
RT-LtR	CGTCTCCTTGACCACCTGCT	
RT-Act LtF	GGAGATGAGGCACAGTCG	Amplification of the actin gene
RT-Act LtR	GCGGTGGTGGAGAAAGAGT	

For the promoter, all conditions were the same as for the coding region, except that the extension time was 15 s. With a Tolo PCR Clean-Up Kit (Tolo Biotech Co., LTD, Shanghai, China), PCR products were purified. The PCR fragment was ligated into the p ESI-blunt vector (YEASEN Biotech Co. Ltd) and sequenced with vector primers M13F and M13R at Tianyihuiyuan Biotech Co., Ltd (Guangzhou, China).

The *LtCYP51* DNA sequence was studied using the programs DNAMAN (version 6.0; LynnonBiosoft, U.S.A.) and InterPro Scan (http://www.ebi.ac.uk/interpro/search/sequence-search), and it was compared *LtCYP51* genes of other fungi. The amino acid sequences of the *LtCYP51* genes from difenoconazole-resistant and sensitive isolates were compared using the computer program EMBOSS Transeq (https://www.ebi.ac.uk/emboss/transeq/), which translated the DNA sequences into amino acid sequences using standard code.

### Quantitative expression of *LtCYP51* gene

Two sensitive isolates (YC70 and JS10) and six resistant isolates (LD10, SY31, YZ90, DZ11, SY05 and YC80) were randomly selected to study the expression of the *LtCYP51* gene. The EC_50_ values of 8 isolates see Table 2. Five mycelial plugs were transferred into a flask containing 100 mL of potato dextrose broth (PDB) and incubated at 28 °C for 24 h on a rotary shaker at 150 rpm. Three flasks were treated with difenoconazole to reach a final concentration of 150 µg/mL, with three replicates. Three flasks containing 100 mL of PDB were used as untreated controls. After being treated with difenoconazole for 12 h, the mycelia of each isolate were removed for RNA extraction^16^. Total RNA was extracted using the TIANGEN RNA simple Total RNA kit (Tiangen Biotech Co., Ltd, Beijing, China), and cDNA was synthesized using the HiScript III 1st Strand cDNA Synthesis Kit with g DNA Eraser (Vazyme Biotech Co., Ltd, Nanjing, China) following the manufacturer's instructions. Every reaction had three biological replicates and three technological replicates.

**Table 2:** The EC_50_ values of *Lasiodiplodia theobromae* isolates to seven fungicides.

Isolates	EC_50_ ± SD (µg/mL)^a^						
	Dif^b^	Car	Pyr	Flu	Bro	Ipr	Teb
YC80	7.59 ± 0.23	8016.29 ± 658.34	1913.83 ± 158.46	0.04 ± 0.01	11.68 ± 1.92	0.30 ± 0.06	0.97 ± 0.11
SY06	9.63 ± 0.66	3.37 ± 0.28	1.75 ± 0.25	0.08 ± 0.01	9.52 ± 1.27	0.53 ± 0.09	1.87 ± 0.13
LD13	1.99 ± 0.22	0.02 ± 0.01	344.93 ± 45.72	0.17 ± 0.02	14.89 ± 1.63	0.36 ± 0.05	0.33 ± 0.08
SY34	10.14 ± 0.91	1792.64 ± 176.28	213.41 ± 19.33	0.04 ± 0.01	10.02 ± 1.15	0.23 ± 0.06	1.11 ± 0.09
YC70	0.97 ± 0.52	0.09 ± 0.01	79 ± 5.38	0.03 ± 0.01	2.08 ± 0.46	0.23 ± 0.07	0.07 ± 0.01
SY05	6.52 ± 0.40	0.38 ± 0.02	260.01 ± 19.62	0.05 ± 0.01	7.83 ± 0.98	0.37 ± 0.08	0.68 ± 0.07
SY31	6.24 ± 0.33	1.08 ± 0.14	83.21 ± 8.45	0.26 ± 0.03	5.04 ± 0.56	0.25 ± 0.09	0.60 ± 0.08
DZ11	8.29 ± 0.36	2.68 ± 0.38	429.48 ± 47.83	0.04 ± 0.01	5.33 ± 0.40	0.40 ± 0.06	0.84 ± 0.09
AM82	1.03 ± 0.85	5447.84 ± 529.11	210.77 ± 20.45	0.11 ± 0.02	7.33 ± 0.72	0.54 ± 0.08	0.17 ± 0.02
YZ31	7.22 ± 0.63	8.39 ± 0.96	133.87 ± 11.23	0.06 ± 0.01	6.46 ± 0.75	0.42 ± 0.03	0.81 ± 0.09
CJ20	2.61 ± 0.57	0.0001 ± 0.00	25.9 ± 1.99	0.11 ± 0.02	8.90 ± 0.66	0.23 ± 0.05	0.47 ± 0.06
YZ01	5.39 ± 0.92	1.17 ± 0.16	8.09 ± 0.93	0.09 ± 0.01	7.36 ± 0.97	0.32 ± 0.04	0.42 ± 0.05
CJ01	0.73 ± 0.33	0.52 ± 0.04	54.02 ± 6.35	0.15 ± 0.02	10.54 ± 0.94	0.30 ± 0.04	0.40 ± 0.07
AM24	2.22 ± 0.30	1.15 ± 0.12	9.98 ± 1.21	0.10 ± 0.01	8.90 ± 0.81	0.25 ± 0.16	0.40 ± 0.06
SY26	8.53 ± 1.17	1.05 ± 0.10	6.07 ± 0.78	0.21 ± 0.03	6.07 ± 0.83	0.35 ± 0.19	0.98 ± 0.08
LD34	0.33 ± 0.29	0.68 ± 0.05	16.58 ± 1.75	0.08 ± 0.01	16.58 ± 1.74	0.42 ± 0.05	0.89 ± 0.09
LD10	6.64 ± 0.41	8537.14 ± 721.73	230.67 ± 20.65	0.12 ± 0.02	9.16 ± 0.95	0.25 ± 0.07	0.63 ± 0.09
SY02	11.56 ± 0.72	2.63 ± 0.45	58.36 ± 4.92	0.17 ± 0.05	8.05 ± 0.75	0.31 ± 0.09	1.59 ± 0.03
JS10	0.65 ± 0.22	1.32 ± 0.23	211.39 ± 19.43	0.05 ± 0.01	8.45 ± 0.92	0.31 ± 0.08	0.07 ± 0.01
YZ90	9.34 ± 0.20	5.59 ± 0.46	0.0008 ± 0.00	0.03 ± 0.01	5.59 ± 0.63	0.36 ± 0.09	0.98 ± 0.09

^a^Values in a column indicate EC_50_ means ± standard deviation (SD).

^b^*Dif* difenoconazole, *Car* carbendazim, *Pyr* pyraclostrobin, *Flu* fludioxonil, *Bro* bromothalonil, *Ipr* iprodione, *Teb* tebuconazole.

Real-time PCR was carried out in a total volume of 20 µL using the qTOWER3 G REAL-TIME PCR thermocycler (Analytik Jena AG, Jena, Germany). To amplify the genes, 2 × ChamQ Universal SYBR qPCR Master Mix (Vazyme Biotech Co., Ltd, Nanjing, China) was employed. The actin gene was amplified as a reference using the primer pair RT-Act LtF/RT-Act LtR to standardize the quantification of *LtCYP51* expression^17^. Three repeats of the experiments were carried out.

### Statistical analysis

The inhibition rates were converted to the probability values, and difenoconazole concentrations were log 10-transformed before using a line regression model. The effective concentration to inhibit mycelial growth by 50% (EC_50_) was calculated by the regression equation. The EC_50_ values were checked for homogeneity of variances using Levene’s test, then the EC_50_ values were calculated for each isolate by combining the data from both replications. The Shapiro–Wilk test was used to determine the normality of the frequency distribution of difenoconazole sensitivity, and the outliers were detected using the boxplot in SPSS 21.0. The histograms were built utilizing log 10-transformed EC_50_ values when the outliers were removed^13,14^. Spearman's rank correlation coefficient using log-transformed EC_50_ values was used to examine cross-resistance among seven fungicides^18,19^. To assess the differences in the relative expression of genes, one-way ANOVA with the LSD test was used (*P* < 0.01). The differences in the mean expression levels were compared by the Mann–Whitney U test (*P* < 0.001). DNAMAN software was used to examine DNA sequences (version 6.0; LynnonBiosoft, U.S.A.).

## Results

### Baseline sensitivity of *L. Theobromae* to difenoconazole

**Figure 1:**
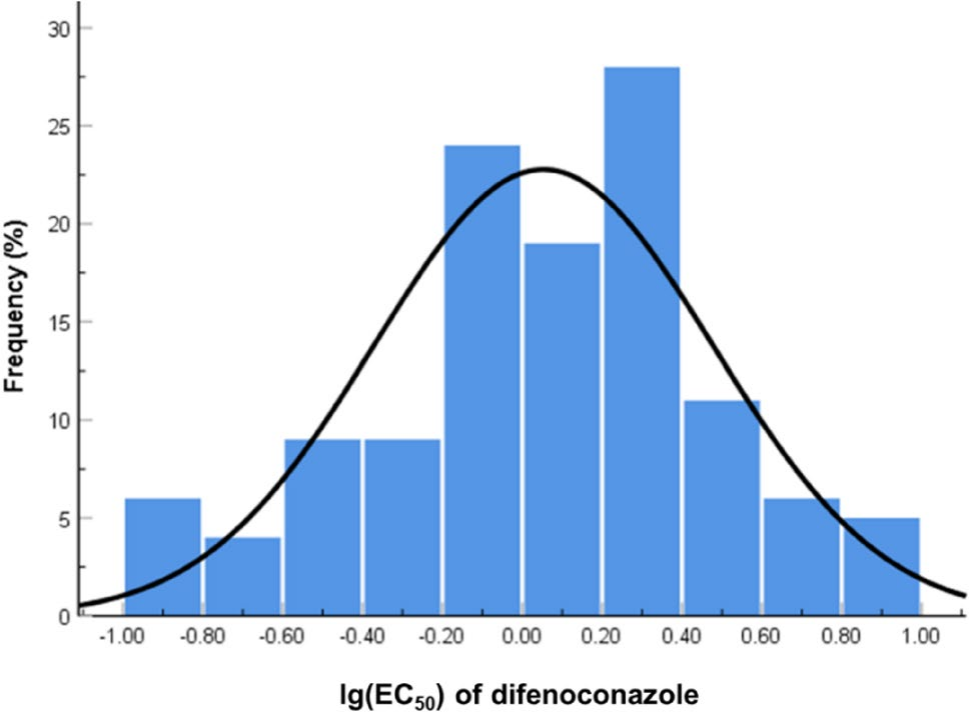
Frequency distribution of lg (EC_50_) values of difenoconazole against 121 *Lasiodiplodia theobromae* isolates when the outliers were excluded.

**Figure 2:**
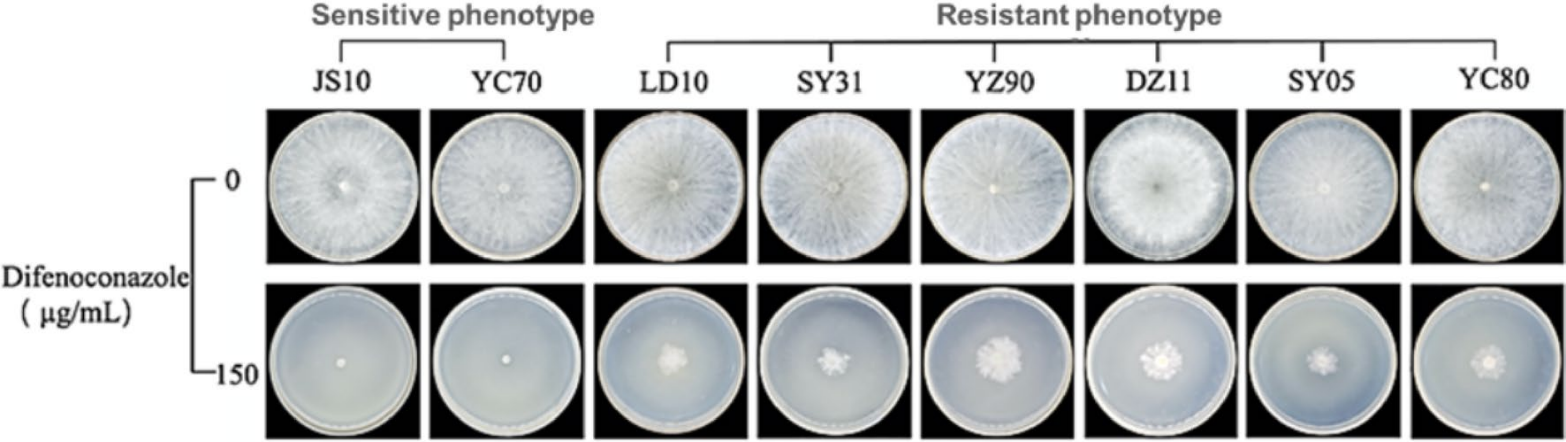
Mycelia colony growth of the eight *Lasiodiplodia theobromae* isolates on PDA plates with and without difenoconazole.

The EC_50_ values of difenoconazole to inhibit mycelial growth of 138 *L. theobromae* field isolates ranged from 0.01 to 13.72 µg/mL. After the outliers were excluded by boxplot, a continuous unimodal log-normal distribution of sensitivity of 121 isolates to difenoconazole was observed (W = 0.981, *P* = 0.087) (Fig. 1). The mean EC_50_ value of 121 isolates was 1.12 ± 1.09 μg/mL, adopted as the resistance threshold concentration. Twenty-one of the 138 isolates were categorized as resistant to difenoconazole based on baseline sensitivity. The EC_50_ values of resistant isolates ranging from 5.60 to 13.72 μg/mL, and the resistance factors ranged from 5 to 12.25. The resistance frequency of *L. theobromae* isolates against difenoconazole was 15.22%. The resistant isolates could grow in the medium containing 150 μg/mL of difenoconazole (Fig. 2).

### Cross-resistance

The EC_50_ of 20 isolates to carbendazim, pyraclostrobin, fludioxonil, bromothalonil, iprodione and tebuconazole were ranged 0.0001–8537.14 μg/mL, 0.0008–1913.83 µg/mL, 0.04–0.26 µg/mL, 2.081–16.58 µg/mL, 0.23–0.54 µg/mL and 0.07–1.87 µg/mL, respectively (Table 2). The results showed that multifungicide-resistant isolates of *L. theobromae* were found. Among 20 isolates used in this study, resistant isolates were resistant to either two (9 isolates), three (1 isolates), or four fungicides (2 isolate).

**Figure 3:**
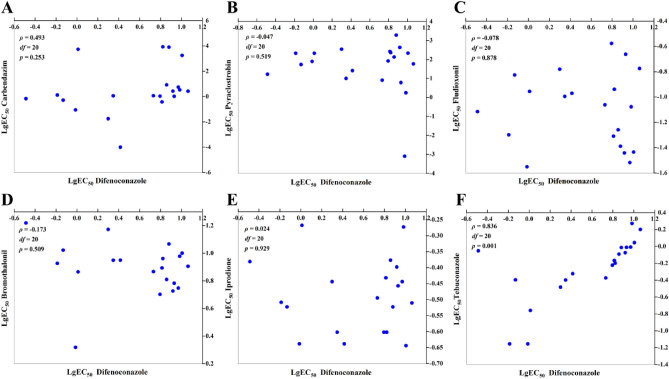
Cross-resistance between difenoconazole and carbendazim (**A**), pyraclostrobin (**B**), fludioxonil (**C**), bromothalonil (**D**), iprodione (**E**), tebuconazole (**F**) by rank correlation analysis. Data shown in logarithmic values of EC_50_ among *Lasiodiplodia theobromae* for fungicide combinations.

There was no correlation between sensitivity to difenoconazole and that to carbendazim (ρ = 0.493, *P* = 0.253; Fig. 3A), pyraclostrobin (ρ = − 0.047, *P* = 0.519; Fig. 3B), fludioxonil (ρ = − 0.078, *P* = 0.878; Fig. 3C), bromothalonil (ρ = − 0.173, *P* = 0.509; Fig. 3D), iprodione (ρ = 0.024, *P* = 0.929; Fig. 3E). Only a positive correlation was observed between sensitivity to difenoconazole and that to tebuconazole (ρ = 0.836, *P* = 0.001; Fig. 3F).

### Cloning and characterization of the *LtCYP51*

The nucleotide sequences of the 1797 bp fragment of the *LtCYP51* gene from the isolates were found to be 99% identical to that of *L. theobromae* (GenBank accession number MK107983.1). The *LtCYP51* gene fragment encodes 523 amino acids and has two introns of 49 bp each at nucleotide positions 247 and 494, respectively. The BLAST search amino acid sequence of the LtCYP51 protein also showed 100%, 94.5% and 93.1% identity with that of the CYP51 protein in *L. theobromae* from cacao (XP_035367211.1), *Diplodia seriata* from grape (OMP84122.1) and *Botryosphaeria dothidea* from apple (KAF4310083.1), respectively.

### Comparison of the *LtCYP51* gene and its upstream region in sensitive and resistant isolates

**Figure 4:**
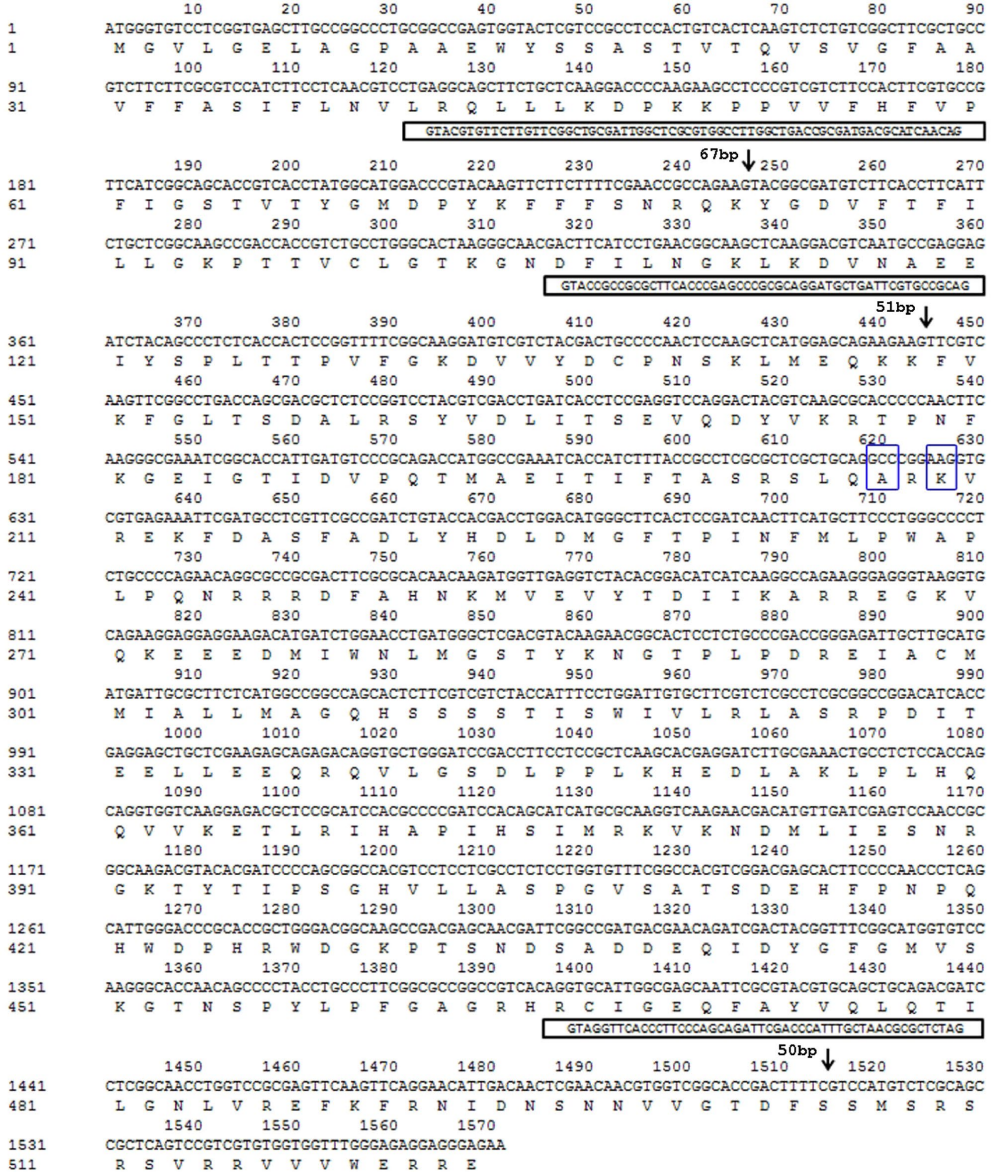
Partial sequences and deduced amino acid sequences of the *LtCYP51* gene from sensitive *Lasiodiplodia theobromae* isolates YC70 (Accession number: MZ365052).The intron sequence is depicted in a solid line box with an arrow showing the insertion site. Two amino acid substitutions were found at position 207 and 209 (in blue box).

The 30 isolates were analyzed for the sequence of *LtCYP51* genes and their upstream regions. Based on the alignment, two mutant phenotypes were found. Compared with other isolates, the sensitive isolate YC70 has two amino acid substitutions at positions 207 (from glycine to alanine, G207A) and 209 (from glutamic acid to lysine, E209k) on the LtCYP51 protein. Furthermore, the resistant isolate YC80 has one amino acid substitution at position 209 (from glutamic acid to lysine, E209k) on the LtCYP51 protein, and this substitution was consistent with the sensitive isolate YC70 (Fig. 4). In the other resistant isolates, no mutation was found.

Fragments approximately 500 bp upstream of the *LtCYP51* gene were obtained using the primer pair Per-1F/Per-1R. The upstream regions were identical in all tested isolates. In any of the isolates tested, no mutations or insertions were identified in the promoter of the *LtCYP51* gene.

### Relative expression of *LtCYP51* in sensitive and resistant isolates

**Figure 5:**
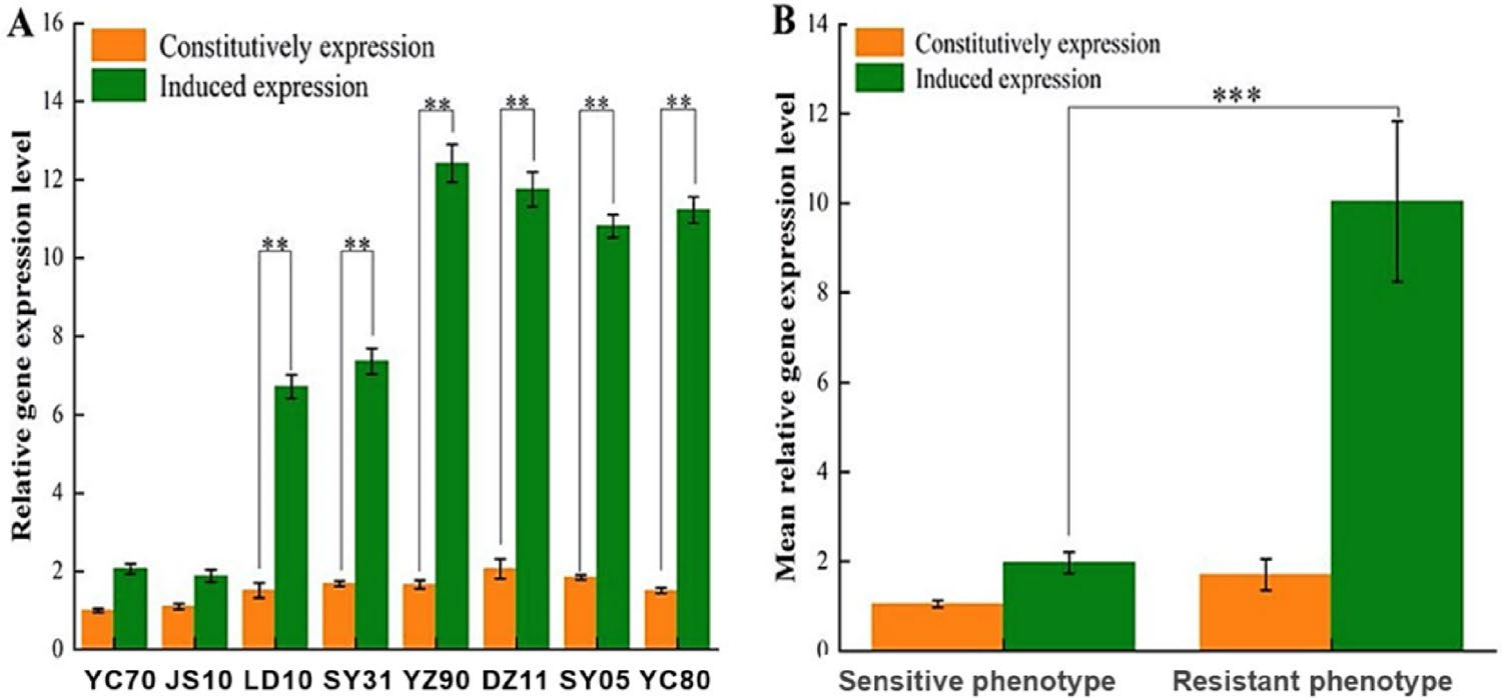
Expression of *Ltcyp51* in the sensitive and resistant isolates of *Lasiodiplodia theobromae* before and after treated by difenoconazole*.* (**A**) Changes of relative expression levels of 8 isolates; (**B**) changes of the mean relative expression levels of different phenotype. **represent significant level (*P* < 0.01), ***represents significant level (*P* < 0.001).

To explore the mechanism of resistance, the expression levels of the *LtCYP51* gene in resistant and sensitive isolates were tested. Our results showed that difenoconazole significantly induced *LtCYP51* expression in the resistant isolates (P < 0.01) (Fig. 5A). The mean constitutive relative expression levels of *LtCYP51* without fungicide in the sensitive and resistant isolates were 1.05 and 1.7 times higher, respectively. Difenoconazole increased the relative expression of *LtCYP51* by 1.87–2.06 times in two sensitive isolates with an average of 1.97, but 6.71–12.41 times in six resistant isolates with an average of 10.05 times. In the resistant isolates, the mean relative expression of *LtCYP51* induced by difenoconazole was fivefold higher than that of sensitive isolates, and this difference was significant (P < 0.001) (Fig. 5B).

## Discussion

Mango diseases are widely controlled using site-specific systemic fungicides in almost all mango-growing regions in the world. The detection of fungicide resistance is a crucial step in monitoring and regulating the spread of resistance in the field^20^. DMI fungicides are classified as a medium risk for resistance development by the Fungicide Resistance Action Committee^21^. DMIs fungicides were more favoured by orchardist due to their specific mode of action and broad anti-fungi spectrum at present. However, DMI resistance has been found in a variety of phytopathogenic fungi^16,17,20^. The resistance mechanisms of DMIs have been reported to be diverse: (I) point mutations in the target gene 14α-demethylase (CYP51)^22–24^; (II) *CYP51* gene overexpression^16,25–29^; and (III) overexpression of efflux proteins^30,31^. In this study, we established the baseline sensitivity of *L. theobromae* to difenoconazole using 121 isolates from five major mango-producing regions in Hainan, China. The results showed that the EC_50_ values ranged from 0.01 to 13.72 µg/mL, with a mean EC_50_ value of 1.1 µg/mL, suggesting that this method could be used as a criterion to judge difenoconazole resistance in further studies. Twenty-one difenoconazole-resistant isolates were found in this study; their EC_50_ values ranged from 5.61 to 13.72 μg/mL. Among systemic fungicides, MBC fungicides are inhibitors of tubulin biosynthesis, which impedes cell division and inhibits mycelial growth^32^. MBC-resistant populations of *L. theobromae* have been confirmed from papaya, citrus and mango^11,33–36^. Our lab discovered that the resistance frequencies of *L. theobromae* isolates to carbendazimmany were more than 70%, and the highly resistant isolates grew normally with 1000 μg/mL carbendazim. Point mutations in the target gene *β-tubulin* were identified in resistant isolates^34^. Compared with carbendazim resistance, DMI resistance of *L. theobromae* from mango is not severe in Hainan. However, the EC_50_ values of 79 isolates to difenoconazole were above 1 µg/mL, which accounted for more than half of the isolates. Thus, a large number of isolates had reduced sensitivity to difenoconazole. Meanwhile, resistant isolates showed positive cross-resistance to difenoconazole and tebuconazole. In addition, no cross-resistance was discovered in this investigation between DMI and non-DMI fungicides. This is consistent with the reported results of *Botrytis cinerea* and *Colletotrichum gloeosporioides* resistance to DMIs^16,37^. In the experiment of cross resistance, we found that there were a small number of isolates with multifungicide resistance (MFR). The result shows that there may be more MFR isolates of *L. theobromae* in fields*.* So it is necessary to continuously detect multifungicide resistance in the future. The commonly used site-specific fungicides (such as carbendazim and azoxystrobin) gave bad control effect against *L. theobromae* due to the development of resistant isolates. With frequent applications of DMI fungicides in fields, the development of DMI fungicide resistance is a major challenge for effective disease control of fruit in China. A appropriate management strategies for fungicide resistance and better management of SER have been suggested, such as reducing the usage of DMI fungicides by combining with alternative fungicides with distinct modes of action that have not been found to cause cross-resistance. For the control of *L. theobromae*, mixtures of difenoconazole and other chemical fungicides, as well as the botanical fungicide Thymol, have been reported to be particularly effective^38^. This management can reduce pathogen population selection pressure, slowing the development of DMI resistance.

The target site of action of DMIs is the enzyme CYP51. The function of *CYP51* is to remove the 14-methyl group of the sterol precursor. DMI fungicides interact with *CYP51* to inhibit the demethylation of lanosterol and influence the production of ergosterol, destroying the integrity and fluidity of the fungal cell membrane. The use of DMI fungicides interfered with ergosterol synthesis activating a *CYP51* response. The function of *CYP51* has been verified in many pathogens^39–42^. DMI fungicides significantly induce *CYP51* expression. Fan et al. proved that *CYP51* gene deletion mutants of *Fusarium graminearum* increased the sensitivity to DMI fungicides prochloraz and difenoconazole^42^. Point mutations of the *CYP51* gene change the conformation of the target protein, resulting in a decrease in the binding ability of fungicides to the target protein. In this study, two amino acid substitutions, E209K and G207A, were found in *LtCYP51* in the sensitive isolate YC70, and one of those amino acid substitutions, E209K, was also found in the resistant isolate YC80. We infer that point mutation of the *CYP51* gene of *L. theobromae* may not be the cause of low-level resistance to difenoconazole. Our studies have reported overexpression of *LtCYP51* in resistant isolates after treatment with difenoconazole. Although most research studies claim that target site changes cause resistance in the majority of DMI-resistant isolates, additional resistance mechanisms independent of *CYP51* mutations cannot be ruled out. Some studies found that the *CYP51* genes of DMI-resistant isolates of plant pathogenic fungi have various point mutations ^42–44^. Studies have also shown that an increase in DMI fungicide application dosages would not improve their efficacy in the case of gene mutations. Some studies reported mutations and overexpression of the *CYP51* gene simultaneously in some DMI-resistant isolates of plant pathogens^17,20,45^. Furthermore, in *L. theobromae* of papaya, *Mycosphaerella graminicola* of wheat, *Blumeriella jaapii* of cherry and *Neophysopella meliosmae-myrianthae* of grapevine no *CYP51* gene point mutation linked to DMI resistance has been identified, but *CYP51* overexpression has been observed^18,30,46,47^. A similar pattern of results was obtained in this study. The results of the present study, do not clearly explain the mechanisms leading to *CYP51*-independent resistance. Regarding the mechanism of *CYP51* overexpression, overexpression of *CYP51* caused by promoter insertions or retrotransposons has only been confirmed in a few phytopathogenic fungi thus far; for example, in *Penicillium digitatum*, increased expression was due to a 199-bp sequence duplication at the promoter of *CYP51*^48^. The mobile genetic element ‘Mona’ is believed to facilitate overexpression of *CYP51* in Monilinia fructicola^49,50^. According to the report of Rallos, overexpression of *CYP51* was associated with the presence of the Y136F mutant genotype^45^. However, the underlying mechanisms of *CYP51* overexpression are not known for in the field DMI-resistant subpopulations of *Puccinia triticina*^43^, *Sclerotinia homoeocarpa*^51^, *Pyrenophora teres*^17^, *Colletotrichum gloeosporioides*^16^, and *Botrytis cinerea*^37^. We tried to clone and sequence analysis the promoter of *LtCYP51* in this study. However, the promoter of *LtCYP51* from difenoconazole-resistant *L. theobromae* isolates did not show any mutations or insertions. The molecular mechanism of *LtCYP51* overexpression needs further investigation by obtaining the complete sequence of the promoter region.

In brief, DMIs have diminished sensitivity in field populations due to their long-term and intensive use. Our results suggest a potential risk for DMI resistance development in *L. theobromae*. In the mango fields of China, Hainan Province, *L. theobromae* has acquired a low to moderate difenoconazole resistance. Although there existed obvious positive cross resistance between difenoconazole and tebuconazole, no cross-resistance was found between difenoconazole and non-DMI fungicides. Control measures such as rotation and mixture treatments with different modes of action fungicides can reduce the emergence of resistant isolates in the field. This means that isolates with multifungicide resistance will become more frequent over time. Compared with the difenoconazole-sensitive isolates, there were no mutations in the *CYP51* gene of resistant isolates at positions 132 or 137 or at any other positions (markers for resistance in DMI fungicides). However, induced expression of *CYP51* in resistant isolates is involved in resistance to difenoconazole. In the future, more research should focus on exploring the mechanisms that induce *CYP51* expression, investigating nontarget site mechanisms of fungicide resistance and the mechanisms of multifungicide resistance in *L. theobromae*. Improved knowledge of fungicide resistance evolution and of the molecular mechanisms by which this occurs will be necessary to implement suitable control strategies that will reduce the likelihood of fungicide resistance outbreaks. Our findings are critical for controlling the high-risk pathogen *L. theobromae* and can help to slow down or even prevent the emergence of DMI fungicide resistance.

## References

[1] Xu B, Wu SJ (2021). Preservation of mango fruit quality using fucoidan coatings. LWT Food. Sci. Technol..

[2] China Industrial Information Network. Analysis on mango varieties, planting area, yield pattern in Hainan province in 2019 and development counter measures in 2020. https://www.chyxx.com/industry/202012/913659.html (2020).

[3] Wang, M. *et al*. Fungicide-resistance and genetic diversity of *Botryodiplodia theobromae* from mango causing stem-end rots in fruits in Hainan. *Chin. J. Tropl. Crop.* **37,** 1363–1369. http://en.cnki.com.cn/Article_en/CJFDTOTAL-RDZX201607019.htm (2016).

[4] Baltazari A (2019). Evaluation of post-harvest losses and shelf life of fresh mango (*Mangifera indica* L.) in eastern zone of Tanzania. Int. J. Fruit. Sci..

[5] Feygenberg O (2014). Improved management of mango fruit though orchard and packinghouse treatments to reduce lenticel discoloration and prevent decay. Postharvest. Biol. Tec..

[6] Galsurker O (2020). Harvesting mango fruit with a short stem-end altered endophytic microbiome and reduce stem-end rot. Microorganisms.

[7] Rodríguez-Gálvez E, Hilário S, Lopes A, Alves A (2020). Diversity and pathogenicity of *Lasiodiplodia* and *Neopestalotiopsis* species associated with stem blight and dieback of blueberry plants in Peru. Eur. J. Plant. Pathol..

[8] Santos PHD (2020). Is *Lasiodiplodia theobromae* the only species that causes leaf blight disease in Brazilian coconut palms?. Trop. Plant. Pathol..

[9] Chen FP (2020). Reduced sensitivity of azoxystrobin and thiophanate-methyl resistance in *Lasiodiplodia theobromae* from papaya. Pestic. Biochem. Phys..

[10] Zhang S (2018). Lasiodiplodia theobromae (Pat.) Griff. & Maubl. reduced energy status and ATPase activity and its relation to disease development and pericarp browning of harvested longan fruit. Food. Chem..

[11] Pereira AV, Martins RB, Michereff SJ, Silva MB, Câmara MPS (2012). Sensitivity of *Lasiodiplodia theobromae* from Brazilian papaya orchards to MBC and DMI fungicides. Eur. J. Plant. Pathol..

[12] Shah MUD, Verma KS (2017). In vitro evaluation of fungitoxicants against *Botryodiplodia theobromae* isolates causing die-back of pear and mango. SKUAST. J. Res..

[13] Yang Y (2021). Multifungicide resistance profiles and biocontrol in *Lasiodiplodia theobromae* from mango fields. Crop. Prot..

[14] Price PP (2015). Fungicide resistance in *Cercospora kikuchii*, a soybean pathogen. Plant. Dis..

[15] FAO. Recommended methods for the detection and measurement of resistance of agricultural pests to pesticides. *FAO. Plant. Prot. Bull.* **2,** 39–42. https://agris.fao.org/agrissearch/search.do?request_locale=ar&recordID=XF19800529554 (1982).

[16] Wei LL (2020). Mutations and overexpression of *CYP51* associated with dmi-resistance in *colletotrichum gloeosporioides* from chili. Plant. Dis..

[17] Mair WJ (2016). Demethylase inhibitor fungicide resistance in *Pyrenophora teres* f.sp. teres associated with target site modification and inducible overexpression of CYP51. Front. Microbiol..

[18] Li Y (2019). Characterization of difenoconazole resistance in *Lasiodiplodia theobromae* from papaya in Brazil. Pest. Manage. Sci..

[19] Sedgwick P (2014). Spearman’s rank correlation coefficient. Br. Med. J..

[20] Zhang C (2020). Two point mutations on *cyp51* combined with induced expression of the target gene appeared to mediate pyrisoxazole resistance in *Botrytis cinerea*. Front. Microbiol..

[21] FRAC Code List. Fungicide sorted by mode of action. http://www.frac.info/docs/default-source/publications/frac-code-list/frac_code_list_2018-final.pdf?sfvrsn=6144b9a_2 (2018).

[22] Chowdhary A (2012). Isolation of multiple-triazole-resistant *Aspergillus fumigatus* strains carrying the TR/L98H mutations in the *cyp51A* gene in India. J. Antimicrob. Chemoth..

[23] Schmitz HK, Medeiros CA, Craig IR, Stammler G (2014). Sensitivity of *Phakopsora pachyrhizi* towards quinone-outside-inhibitors and demethylation-inhibitors and corresponding resistance mechanisms. Pest. Manage. Sci..

[24] Wang F (2015). The Y137H mutation of *VvCYP51* gene confers the reduced sensitivity to tebuconazole in *Villosiclava virens*. Sci. Rep..

[25] Cools HJ, Bayon C, Atkins S, Lucas JA, Fraaije BA (2012). Overexpression of the sterol 14α-demethylase gene (*MgCYP51*) in *Mycosphaerella graminicola* isolates confers a novel azole fungicide sensitivity phenotype. Pest. Manage. Sci..

[26] Carter HE (2014). Alterations in the predicted regulatory and coding regions of the sterol 14α-demethylase gene (*CYP51*) confer decreased azole sensitivity in the oilseed rape pathogen *Pyrenopeziza brassicae*. Mol. Plant. Pathol..

[27] Hulvey J, Popko JT, Sang H, Berg A, Jung G (2012). Overexpression of Sh CYP51B and Shatr D in *Sclerotinia homoeocarpa* isolates exhibiting practical field resistance to a demethylation inhibitor fungicide. Appl. Environ. Microb..

[28] Nikou D (2009). Molecular characterization and detection of overexpressed C-14 alpha-demethylase-based DMI resistance in *Cercospora beticola* field isolates. Pestic. Biochem. Phys..

[29] Rodriguez-Tudela JL (2008). Epidemiological cutoffs and cross-resistance to azole drugs in *Aspergillus fumigatus*. Antimicrob. Agents. Ch..

[30] Leroux P, Walker AS (2011). Multiple mechanisms account for resistance to sterol 14α-demethylation inhibitors in field isolates of *Mycosphaerella graminicola*. Pest. Manage. Sci..

[31] Omrane S (2015). Fungicide efflux and the Mg MFS1 transporter contribute to the multidrug resistance phenotype in *Zymoseptoria tritici* field isolates. Environ. Microbiol..

[32] David VC, Diego R, Antonio V, Alejandro PG (2018). Analysis of β-tubulin-carbendazim interaction reveals that binding site for MBC fungicides does not include residues involved in fungicide resistance. Sci. Rep..

[33] Rehman A (2015). Emerging resistance against different fungicides in *Lasiodiplodia theobromae* as the cause of mango dieback in Pakistan. Arch. Biol. Sci..

[34] Yang Y, Zeng GD, Zhang Y, Xue R, Hu YJ (2019). Molecular and biochemical characterization of carbendazim-resistant *Botryodiplodia theobromae* field isolates. Plant. Dis..

[35] Zhang JX, Timmer LW (2006). Preharvest application of fungicides for postharvest disease control on early season tangerine hybrids in Florida. Crop. Prot..

[36] Zhao, L., Yang, Y., Wang, M., He, R. & Chen, M. C. Sensitivities of carbendazim-resistance and fitness of *Botryodiplodia theobromae* isolates from mango in Hainan. *Chin. J. Pestic. Sci*. **19,** 1–9. http://en.cnki.com.cn/Article_en/CJFDTOTAL-NYXB201703005.htm (2017).

[37] Zhang C (2020). Difenoconazole resistance shift in *Botrytis cinerea* from tomato in china associated with inducible expression of CYP51. Plant. Dis..

[38] Ye, H. C., Zhou, Y., Zhang, J., Yan, C. & Feng, G. Synergistic toxicity of thymol and difenoconazole on mango pathogenic fungi *Botryodiplodia theobroma*. *Chin. J. Trop. Agric*. **35,** 89–93. http://en.cnki.com.cn/Article_en/CJFDTOTAL-RDNK201512017.htm (2015).

[39] Xin L (2011). Paralogous *cyp51* genes in *Fusarium graminearum* mediate differential sensitivity to sterol demethylation inhibitors. Fungal. Genet. Biol..

[40] Fan J (2013). Characterization of the sterol 14α-demethylases of *Fusarium graminearum* identifies a novel genus-specific *CYP51* function. New. Phytol..

[41] Muellender MM, Mahlein AK, Stammler G, Varrelmann M (2020). Evidence for the association of target-site resistance in *cyp51* with reduced DMI sensitivity in European *Cercospora beticola* field isolates. Pest. Manage. Sci..

[42] Fan J (2014). The Y123H substitution perturbs *FvCYP51B* function and confers prochloraz resistance in laboratory mutants of *Fusarium verticillioides*. Plant. Pathol..

[43] Stammler G, Cordero J, Koch A, Semar M, Schlehuber S (2009). Role of the Y134F mutation in *CYP51* and overexpression of *CYP51* in the sensitivity response of *Puccinia triticina* to epoxiconazole. Crop. Prot..

[44] Chen FP (2015). Heterologous expression of the *Monilinia fructicola CYP51* (*MfCYP51*) gene in *Pichia pastoris* confirms the mode of action of the novel fungicide, SYP-Z048. Front. Microbiol..

[45] Rallos LEE, Baudoin AB (2016). Co-occurrence of two allelic variants of *CYP51* in Erysiphe necator and their correlation with over-expression for DMI resistance. PLoS One.

[46] Ma ZH, Proffer TJ, Jacobs JL, Sundin GW (2006). Overexpression of the 14α-demethylase target gene (*CYP51*) mediates fungicide resistance in *Blumeriella jaapii*. Appl. Environ. Microb..

[47] Santos RF, Amorim L, Wood AKM, Bibiano LB, Fraaije B (2021). Lack of an intron in cytochrome b and overexpression of sterol 14α-demethylase indicate a potential risk for QoI and DMI resistance development in *Neophysopella* spp. on grapes. Phytopathology.

[48] Sun, X. P., Wang, J. Y., Feng, D., Ma, Z. H. & Li, H. Y. *PdCYP51B*, a new putative sterol 14α-demethylase gene of *Penicillium digitatum* involved in resistance to imazalil and other fungicides inhibiting ergosterol synthesis. *Appl. Microbiol. Biot*. 10.1007/s00253-011-3355-7 21637936

[49] Luo C, Cox KD, Amiri A, Schnabel G (2008). Occurrence and detection of the DMI resistance-associated genetic element ‘Mona’ in *Monilinia fructicola*. Plant Dis..

[50] Villani SM, Cox KD (2011). Characterizing fenbuconazole and propiconazole sensitivity and prevalence of ‘Mona’ in isolates of *Monilinia fructicola* from New York. Plant Dis..

[51] Ma B, Tredway LP (2013). Induced overexpression of cytochrome P450 sterol 14alpha-demethylase gene (*CYP51*) correlates with sensitivity to demethylation inhibitors (DMIs) in *Sclerotinia homoeocarpa*. Pest. Manage. Sci..

